# Supplementary material for: Unsupervised Clustering of Patients Undergoing Thoracoscopic Ablation Identifies Relevant Phenotypes for Advanced Atrial Fibrillation
Source: Diagnostics (Basel). 2025 May 16;15(10):1269. doi: 10.3390/diagnostics15101269 (PMC12110638; doi:10.3390/diagnostics15101269)
Supplement: Supplementary file 1 [file diagnostics-15-01269-s001.zip › SupplementaryMaterials.pdf]

Supplementary Materials

Unsupervised dimensionality reduction analysis

We plotted the variables that had the largest weight in the composition of each principal component. Subsequently, we plotted AF type, CHA_2DS_2-VASc score and the treatment outcome variables, namely 1-year and 2-year AF recurrence. We visually analyzed their separation and calculated the silhouette scores for the nominal categorical variables. The silhouette score is a metric that quantifies the separation of clusters by comparing the average distance within each cluster to the distance between different clusters. A silhouette score with a value of minus one indicates no separation, whereas a value of one indicates strong clustering. A silhouette score close to zero means that the groups are separated, but the boundaries of the groups are adjacent or overlap. Since this score does not incorporate the disposition of the clusters, we only performed a visual separation analysis for the CHA_2DS_2-VASc score.

Supplementary Figure S2 shows the variables with the largest impact on the principal components. The first principal component was composed of variables related to the presence of AF before and at the start of the procedure, left atrial morphology, and pro-brain natriuretic peptide value (common biomarker of heart failure). The second component included blood pressure, hypertension, age, and cholesterol as main factors. The third component was composed of the heart rate, QRS complex, pulmonary vein ostium height and left atrial morphology.

Supplementary Figure S3 displays clinically relevant phenotypes, in particular AF type and CHA_2DS_2-VASc score. AF type was visually separated along the first principal component (Supplementary Figure S3A-C). The silhouette score of the paroxysmal AF patients compared to the (longstanding) persistent AF patients is 0.03, indicating that the

boundaries of the two groups lie close together, but that the groups are separated. The CHA_2DS_2-VASc scores were separated along the second and third principal components (Supplementary Figure S3D-F).

AF recurrence outcome is visually separated along the first principal component and the third component (Supplementary Figure S4). Both the silhouette scores of 1-year and 2-year AF recurrence were 0.01. Thus, the groups with and without AF recurrence are separated, but the boundaries of the groups overlap in the lower dimensional space.

Tables & Figures

Table S1: Distribution of all included clinical variables in the original dataset, the dataset without outliers and the imputed dataset.

	**Original**	**Outliers removed**	**Imputed**	**p-value**
General				
Age (y)	61 (55-67)	61 (55-67)	61 (56-66)	0.94
Female - n (%)	169 (26%)	169 (26%)	169 (26%)	0.99
AF type - n (%)				1.0
Paroxysmal	234 (35%)	231 (35%)	231 (35%)	
Persistent	414 (62%)	409 (62%)	409 (62%)	
Longstanding persistent	18 (3%)	18 (3%)	18 (3%)	
BMI (kg/m2)	27.2 (24.9-30.2)	27.3 (24.9-30.2)	27.4 (24.9-30.2)	0.99
Total duration of AF (y)	4 (2-8)	4 (2-8)	4 (2-8)	1.0
Systolic blood pressure (mmHg)	132 (120-145)	132 (120-145)	132 (120-145)	0.99
Smoking - n (%)	93 (14%)	93 (14%)	93 (14%)	0.09
History				
Other cardiac diseases - n (%)	41 (6%)	41 (6%)	41 (6%)	0.93
Previous catheter PVI - n (%)	120 (18%)	120 (18%)	120 (18%)	1.0
Pacemaker implantation - n (%)	23 (3%)	23 (3%)	23 (3%)	1.0
Total electrical cardioversions	2 (1-5)	2 (1-5)	3 (1-4)	0.07
CHADSVASc				
CHA2DS2-VASc - n (%)				1.0
0	169 (25%)	165 (25%)	165 (25%)	
1	219 (33%)	215 (33%)	215 (33%)	

2	156 (23%)	155 (24%)	155 (24%)	
3	77 (12%)	76 (12%)	76 (12%)	
4	28 (4%)	28 (4%)	28 (4%)	
5	10 (1%)	10 (2%)	10 (2%)	
6	8 (1%)	8 (1%)	8 (1%)	
7	1 (0%)	1 (0%)	1 (0%)	
Congestive heart disease - n (%)	45 (7%)	45 (7%)	45 (7%)	1.0
Diabetes mellitus - n (%)	43 (7%)	43 (7%)	43 (7%)	1.0
Hypertension - n (%)	291 (44%)	291 (44%)	291 (44%)	1.0
Stroke/TIA/Embolism - n (%)	52 (8%)	52 (8%)	52 (8%)	1.0
Vascular disease - n (%)	87 (13%)	87 (13%)	87 (13%)	1.0
ECG				
AV block - n (%)	84 (13%)	84 (13%)	84 (13%)	0.96
Heart rate (bpm)	64 (55-78)	64 (55-78)	64 (56-78)	0.98
QRS (ms)	98 (88-106)	98 (88-106)	98 (88-106)	1.0
QTc (ms)	429 (411-448)	429 (411-448)	429 (412-447)	1.0
Rhythm - n (%)				1.0
Sinus Rhythm	340 (53%)	334 (52%)	352 (53%)	
Sinus Bradycardia	120 (19%)	120 (19%)	120 (18%)	
Sinus Tachycardia	4 (1%)	4 (1%)	4 (1%)	
Atrial Rhythm	3 (0%)	3 (0%)	3 (0%)	
Atrial Tachycardia	2 (0%)	2 (0%)	2 (0%)	
Atrial Flutter	14 (2%)	14 (2%)	14 (2%)	
AF	163 (25%)	163 (25%)	163 (25%)	
Ventricular conduction - n (%)	45 (7%)	45 (7%)	45 (7%)	0.96
Holter				
Atrial flutter - n (%)	76 (12%)	76 (12%)	76 (12%)	0.87
Atrial Tachycardia - n (%)	48 (7%)	48 (7%)	48 (7%)	0.91
AV block - n (%)	109 (17%)	109 (17%)	109 (17%)	0.75
Maximum heart rate (bpm)	126 (101-155)	127 (102-156)	127 (103-154)	0.94
Minimal heart rate (bpm)	47 (41-54)	46 (41-53)	47 (41-53)	0.98
Imaging				
Left atrial volume index (LAVI)	40.0 (33.1-48.8)	40.0 (33.0-48.7)	40.0 (33.4-48.3)	0.99
Pulmonary vein abnormalities - n (%)	74 (11%)	74 (11%)	74 (11%)	0.95
Cardiac abnormalities - n (%)	93 (14%)	93 (14%)	93 (14%)	0.95
Aberrant pulmonary veins - n (%)	86 (13%)	86 (13%)	86 (13%)	0.96

Anterior posterior length (mm)	40.0 (35.0-47.0)	40.0 (35.0-47.0)	40.4 (35.2-47.0)	0.62
Craniocaudal length (mm)	67 (60-72)	67 (60-72)	66 (62-71)	0.56
Height of LSPV ostium (mm)	15 (13-18)	15 (13-18)	15 (13-18)	0.92
Pulmonary vein stenosis - n (%)	3 (0%)	3 (0%)	3 (0%)	0.99
Height of RIPV ostium (mm)	16 (14-19)	16 (14-19)	16 (14-19)	0.97
Height of RSPV ostium (mm)	17 (14-20)	17 (14-20)	17 (15-20)	0.98
Transverse diameter (mm)	73.0 (65.0-80.0)	74.0 (66.0-80.0)	73.7 (67.8-80.0)	0.57
Biomarkers				
Creatinine (µmol/L)	86 (76-98)	86 (76-98)	86 (76-98)	1.0
CRP (mg/L)	1.4 (0.7-2.9)	1.4 (0.7-2.9)	1.8 (0.8-3.3)	**0.02**
Hemoglobine (mmol/L)	9.2 (8.6-9.7)	9.2 (8.6-9.6)	9.2 (8.6-9.6)	0.97
Potassium (mmol/L)	4.3 (4.0-4.5)	4.3 (4.0-4.5)	4.3 (4.0-4.5)	0.99
Leukocytes (10^9/L)	6.8 (5.5-8.0)	6.7 (5.5-7.9)	6.7 (5.6-7.9)	0.97
Sodium (mmol/L)	141.0 (139.0-142.0)	141.0 (139.0-142.0)	141.0 (139.0-142.0)	1.0
ProBNP (ng/L)	314.5 (117.2-705.2)	314.0 (115.0-705.0)	325.0 (128.8-702.0)	0.9
Thrombocytes (10^9/L)	228.0 (195.5-263.0)	228.0 (196.0-263.0)	228.0 (197.0-262.8)	0.99
TSH (mE/L)	2.0 (1.4-2.8)	2.0 (1.4-2.8)	2.1 (1.5-2.9)	0.52
Medication				
Use of ACE inhibitors - n (%)	156 (24%)	156 (24%)	156 (24%)	0.98
Use of ATII inhibitors - n (%)	123 (19%)	123 (19%)	123 (19%)	1.0
Use of calcium-antagonists - n (%)	62 (9%)	62 (9%)	62 (9%)	0.91
Use of cholesterol medication - n (%)	173 (26%)	173 (26%)	173 (26%)	1.0
Use of class IA antiarrhythmic medication - n (%)	14 (2%)	14 (2%)	14 (2%)	0.84
Use of class IC antiarrhythmic medication - n (%)	190 (29%)	190 (29%)	190 (29%)	1.0
Use of class II medication - n (%)	312 (47%)	312 (47%)	312 (47%)	0.99
Use of class III antiarrhythmic medication - n (%)	288 (44%)	288 (44%)	288 (44%)	0.96

Use of class IV antiarrhythmic medication - n (%)	85 (13%)	85 (13%)	85 (13%)	0.99
Use of loop diuretics - n (%)	71 (11%)	71 (11%)	71 (11%)	0.98
Use of nitrates medication - n (%)	14 (2%)	14 (2%)	14 (2%)	0.84
Use of anticoagulation medication - n (%)	641 (97%)	641 (97%)	641 (97%)	1.0
Use of potassium diuretics - n (%)	28 (4%)	28 (4%)	28 (4%)	0.92
Use of thiazide diuretics - n (%)	77 (12%)	77 (12%)	77 (12%)	0.98
Use of a thrombocyte inhibitor - n (%)	27 (4%)	27 (4%)	27 (4%)	0.92
Procedure				
Rhythm start procedure - n (%)				0.15
Sinus	281 (54%)	281 (54%)	396 (60%)	
AF	234 (45%)	233 (45%)	254 (39%)	
Other	8 (2%)	8 (2%)	8 (1%)	
Total of number RPV ablations	8 (7-10)	8 (7-10)	8 (7-10)	0.93
Number of RPV ablation attempts/series	1 (1-1)	1 (1-1)	1 (1-1)	0.85
Total number of LPV ablations	8 (7-10)	8 (7-10)	8 (7-10)	0.98
Number of LPV ablation attempts/series	1 (1-1)	1 (1-1)	1 (1-1)	0.84
LPV isolation - n (%)	632 (96%)	632 (96%)	632 (96%)	0.98
Extended lesions - n (%)	437 (66%)	437 (66%)	437 (66%)	0.97
Trigone line - n (%)				1.0
No	233 (36%)	232 (36%)	238 (36%)	
Yes, not isolated	116 (18%)	116 (18%)	116 (18%)	
Yes, isolated	293 (46%)	291 (46%)	304 (46%)	
Stress test				
Type of arrhythmia - n (%)				1.0
None	392 (63%)	391 (63%)	416 (63%)	
Atrial Tachycardia	5 (1%)	5 (1%)	5 (1%)	
Atrial Flutter	15 (2%)	15 (2%)	15 (2%)	
AF	205 (33%)	205 (33%)	220 (33%)	
NSVT	2 (0%)	2 (0%)	2 (0%)	
Duration exercise test	7.2 (5.5-9.4)	7.2 (5.5-9.4)	7.3 (5.5-9.3)	0.97
Reason of ending - n (%)				1.0

None	23 (4%)	23 (4%)	23 (3%)	
Fatigued	455 (73%)	455 (73%)	492 (75%)	
Chest Pain	5 (1%)	5 (1%)	5 (1%)	
Palpitation	5 (1%)	5 (1%)	5 (1%)	
Leg Pain	60 (10%)	60 (10%)	60 (9%)	
Other	74 (12%)	73 (12%)	73 (11%)	
Ischemia - n (%)	35 (5%)	35 (5%)	35 (5%)	0.95
Minimal heart rate (bpm)	179 (156-200)	180 (158-201)	179 (159-199)	0.82
Minimal systolic RR interval (mmHg)				1.0

*Numerical variables are reported as median (interquartile range). Categorical variables are reported as number (percentage). Numerical variables were tested using the kruskal-wallis test. Categorical variables were tested with the chi-squared test. *:<0.01, **:<0.001,***:<0.0001. L(I/S)PV: left (inferior/superior) pulmonary vein; R(I/S)PV: right (inferior/superior) pulmonary vein; proBNP: pro-brain natriuretic peptide; ATII: angiotensin II; CRP: C-Reactive protein; TSH: Thyroid-stimulating hormone.*

Table S2: Discarded variables and the corresponding reasons for discarding.

Variable	**Reason discarding**
General	
Cardiovascular disease in family	>30% missing values
Patient's history	
Previous cardiac procedure/surgery	>30% missing values
Congestive heart failure	Correlated to congestive heart disease
Myocardial infarction	Correlated to percutaneous coronary intervention
Percutaneous coronary intervention	Correlated to vascular disease
CHADSVASc	
Cholesterol	Correlated to cholesterol medication
ECG	
PR	>30% missing values
QT	Correlated to heart rate
Holter	
AF presence	Correlated to max heart rate
Average heart rate	Correlated to minimal heart rate Holter
Imaging	
Left ventricular ejection fraction	>30% missing values
Aortic valve stenosis	>30% missing values
Mitral valve insufficiency	>30% missing values
Mitral valve stenosis	>30% missing values
Width of LIPV ostium (mm)	>30% missing values
Width of LSPV ostium (mm)	>30% missing values

Width of RIPV ostium (mm)	>30% missing values
Width of RIPV ostium (mm)	>30% missing values
Height of LIPV ostium (mm)	Correlated to height LSPV ostium
Biomarkers	
TropT	>30% missing values
Procedure	
Total number of electrical cardioversions needed	>30% missing values
RPV isolation	Correlated to LPV isolation
Lesions roof	Correlated to extended lesions
Lesions trigone	Correlated to extended lesions
Stress test	
Minimal diastolic RR-interval	>30% missing values
Maximal diastolic RR-interval	>30% missing values
METS	>30% missing values
Watt	>30% missing values
Minimal heart rate	Correlated to average heart rate Holter
Maximal heart rate	Correlated to maximal heart rate Holter
Arrhythmia present	Correlated to AF present Holter
Maximal systolic RR interval	Correlated to minimal systolic RR interval

L(I/S)PV: left (inferior/superior) pulmonary vein; R(I/S)PV: right (inferior/superior) pulmonary vein; TropT: Troponin T.

Table S3: All included clinical variables and their distribution over the three clusters.

	**1 (n = 308)**	**2 (n = 188)**	**3 (n = 162)**	**p-value**	**(1, 2)**	**(1, 3)**	**(2, 3)**
General							
Age (y)	58 (51-64)	62 (56-67)	66 (59-70)	***	***	***	*
Female - n (%)	60 (19%)	54 (29%)	55 (34%)	*	0.07	*	1.05
AF type - n (%)				***	***	**0.01**	***
Paroxysmal	161 (52%)	11 (6%)	59 (36%)				
Persistent	145 (47%)	162 (86%)	102 (63%)				
Longstanding persistent	2 (1%)	15 (8%)	1 (1%)				
BMI (kg/m2)	26.6 (24.2-29.3)	28.1 (25.2-30.9)	27.9 (25.1-31.1)	**	**	*	2.89
Total duration of AF (y)	4 (2-8)	4 (2-9)	5 (2-8)	0.63			
Systolic blood pressure (mmHg)	126 (118-140)	130 (120-142)	145 (131-160)	***	0.2	***	***
Smoking - n (%)	44 (18%)	28 (19%)	21 (18%)	0.91			
Outcome							

AF recurrence after two years - n (%)	112 (39%)	81 (49%)	72 (49%)	0.05			
Antiarrhythmic drug (AAD) use - n (%)	55 (19%)	52 (30%)	49 (32%)	*	**0.03**	**0.01**	2.63
AF recurrence after one year - n (%)	78 (26%)	67 (37%)	55 (35%)	**0.02**	**0.04**	0.18	2.3
Failure type - n (%)				0.14			
None	184 (62%)	91 (51%)	82 (52%)				
Atrial Tachycardia	51 (17%)	43 (24%)	40 (25%)				
Atrial Flutter	12 (4%)	6 (3%)	7 (4%)				
AF	48 (16%)	39 (22%)	28 (18%)				
History							
Other cardiac diseases - n (%)	18 (7%)	12 (6%)	11 (7%)	0.94			
Previous catheter PVI - n (%)	76 (25%)	16 (9%)	28 (17%)	***	***	0.26	0.07
Pacemaker implantation - n (%)	5 (2%)	9 (5%)	9 (6%)	**0.04**	0.21	0.11	2.86
Total electrical cardioversions	2 (1-4)	3 (1-5)	3 (1-7)	0.15			
CHADSVASc							
CHA2DS2-VASc - n (%)				***	***	***	***
0	132 (43%)	33 (18%)	-				
1	114 (37%)	69 (37%)	32 (20%)				
2	48 (16%)	52 (28%)	55 (34%)				
3	9 (3%)	17 (9%)	50 (31%)				
4	5 (2%)	12 (6%)	11 (7%)				
5	-	4 (2%)	6 (4%)				
6	-	1 (1%)	7 (4%)				
7	-	-	1 (1%)				
Congestive heart disease - n (%)	10 (3%)	28 (15%)	7 (4%)	***	***	2.22	*
Diabetes mellitus - n (%)	4 (1%)	11 (6%)	28 (17%)	***	**0.03**	***	*
Hypertension - n (%)	66 (21%)	80 (43%)	145 (90%)	***	***	***	***
Stroke/TIA/Embolism - n (%)	14 (5%)	15 (8%)	23 (14%)	*	0.5	*	0.27
Vascular disease - n (%)	18 (6%)	23 (12%)	46 (28%)	***	0.06	***	**

ECG							
AV block - n (%)	35 (12%)	8 (5%)	41 (26%)	***	**0.04**	**	***
Heart rate (bpm)	58 (52-66)	85 (74-97)	61 (56-69)	***	***	*	***
QRS (ms)	100 (91-108)	94 (84-100)	98 (88-108)	***	***	1.72	**
QTc (ms)	422 (407-440)	438 (417-457)	433 (416-455)	***	***	***	1.12
Rhythm - n (%)				***	***	0.3	***
Sinus Rhythm	199 (66%)	29 (16%)	106 (68%)				
Sinus Bradycardia	84 (28%)	4 (2%)	32 (20%)				
Sinus Tachycardia	1 (0%)	2 (1%)	1 (1%)				
Atrial Rhythm	1 (0%)	1 (1%)	1 (1%)				
Atrial Tachycardia	-	-	2 (1%)				
Atrial Flutter	5 (2%)	6 (3%)	3 (2%)				
AF	10 (3%)	141 (77%)	12 (8%)				
Ventricular conduction - n (%)	21 (7%)	10 (6%)	14 (9%)	0.52			
Holter							
Atrial flutter - n (%)	41 (15%)	17 (9%)	18 (12%)	0.25			
Atrial Tachycardia - n (%)	27 (10%)	2 (1%)	19 (12%)	**	*	1.33	**
AV block - n (%)	53 (19%)	9 (5%)	47 (31%)	***	**	**0.02**	***
Maximum heart rate (bpm)	122 (100-157)	143 (123-160)	107 (89-137)	***	***	**	***
Minimal heart rate (bpm)	45 (39-50)	53 (46-61)	45 (40-49)	***	***	1.32	***
Imaging							
Left atrial volume index (LAVI)	37.1 (30.7-45.0)	43.8 (35.1-54.3)	41.2 (34.9-52.4)	***	***	***	1.23
Aberrant pulmonary veins - n (%)	35 (12%)	29 (16%)	22 (14%)	0.46			
Anterior posterior length (mm)	38 (33-42)	46 (40-51)	42 (38-48)	***	***	***	**
Craniocaudal length (mm)	64 (59-70)	70 (65-76)	67 (60-71)	***	***	0.28	***
Height of LSPV ostium (mm)	15 (12-18)	15 (13-18)	15 (12-18)	0.22			
Pulmonary vein stenosis - n (%)	-	2 (1%)	1 (1%)	0.23			

Height of RIPV ostium (mm)	16 (13-19)	17 (14-19)	16 (14-20)	0.1			
Height of RSPV ostium (mm)	17 (14-20)	18 (15-21)	18 (15-21)	*	*	0.09	1.53
Transverse diameter (mm)	70 (63-77)	79 (73-85)	75 (68-80)	***	***	*	***
Biomarkers							
Creatinine (µmol/L)	85 (75-96)	92 (80-102)	84 (74-95)	**	**	2.3	*
CRP (mg/L)	1.1 (0.6-2.5)	1.9 (0.8-3.6)	1.6 (0.9-3.4)	**	*	**	2.36
Hemoglobine (mmol/L)	9.2 (8.7-9.6)	9.4 (8.7-9.8)	8.9 (8.4-9.4)	***	0.13	*	***
Potassium (mmol/L)	4.2 (4.0-4.5)	4.4 (4.1-4.6)	4.2 (3.9-4.4)	***	*	**0.01**	***
Leukocytes (10^9/L)	6.5 (5.3-7.9)	7.0 (5.8-8.0)	6.8 (5.5-8.0)	**0.04**	**0.03**	0.51	1.13
Sodium (mmol/L)	141 (140-142)	140 (139-142)	141 (139-142)	**0.04**	**0.04**	0.42	1.29
ProBNP (ng/L)	178.0 (85.0-414.0)	802.0 (454.8-1220.5)	240.5 (114.0-476.8)	***	***	0.06	***
Thrombocytes (10^9/L)	230.5 (196.0-262.8)	222.0 (198.0-259.0)	233.5 (194.8-272.0)	0.63			
TSH (mE/L)	2.0 (1.3-2.9)	2.0 (1.5-2.7)	2.1 (1.5-3.0)	0.64			
Medication							
Use of ACE inhibitors - n (%)	41 (13%)	53 (28%)	62 (38%)	***	**	***	0.18
Use of ATII inhibitors - n (%)	25 (8%)	25 (13%)	73 (45%)	***	0.26	***	***
Use of calcium-antagonists - n (%)	10 (3%)	9 (5%)	43 (27%)	***	1.59	***	***
Use of cholesterol medication - n (%)	41 (13%)	39 (21%)	93 (57%)	***	0.12	***	***
Use of class IA antiarrhythmic medication - n (%)	11 (4%)	-	3 (2%)	**0.03**	0.06	1.35	0.59
Use of class IC antiarrhythmic medication - n (%)	127 (41%)	26 (14%)	37 (23%)	***	***	**	0.12

Use of class II medication - n (%)	138 (45%)	107 (57%)	67 (41%)	*	**0.03**	1.61	**0.02**
Use of class III antiarrhythmic medication - n (%)	128 (42%)	66 (35%)	94 (58%)	***	0.55	*	***
Use of class IV antiarrhythmic medication - n (%)	33 (11%)	36 (19%)	16 (10%)	**0.01**	**0.04**	2.7	0.07
Use of loop diuretics - n (%)	12 (4%)	36 (19%)	23 (14%)	***	***	**	0.83
Use of nitrates medication - n (%)	1 (0%)	5 (3%)	8 (5%)	*	0.18	*	1.2
Use of anticoagulation medication - n (%)	297 (96%)	185 (98%)	159 (98%)	0.32			
Use of potassium diuretics - n (%)	3 (1%)	18 (10%)	7 (4%)	***	***	0.12	0.27
Use of thiazide diuretics - n (%)	9 (3%)	13 (7%)	55 (34%)	***	0.18	***	***
Use of a thrombocyte inhibitor - n (%)	11 (4%)	4 (2%)	12 (7%)	**0.04**	1.57	0.32	0.11
Procedure							
Rhythm start procedure - n (%)				***	***	1.23	***
Sinus	162 (74%)	29 (17%)	90 (68%)				
AF	52 (24%)	141 (82%)	40 (30%)				
Other	4 (2%)	2 (1%)	2 (2%)				
Total of number RPV ablations	8 (6-10)	8 (7-11)	8 (7-10)	**	**	0.12	0.31
Number of RPV ablation attempts/series	1 (1-1)	1 (1-1)	1 (1-1)	0.61			
Total number of LPV ablations	8 (7-10)	8 (7-9)	8 (7-10)	0.4			
Number of LPV ablation attempts/series	1 (1-1)	1 (1-1)	1 (1-1)	*	*	0.6	0.2
LPV isolation - n (%)	275 (95%)	174 (97%)	155 (97%)	0.47			

Extended lesions - n (%)	156 (52%)	167 (90%)	103 (64%)	***	***	0.05	***
Superior line - n (%)				***	***	0.05	***
No	142 (49%)	20 (11%)	59 (37%)				
Yes, not isolated	26 (9%)	15 (8%)	10 (6%)				
Yes, isolated	124 (42%)	152 (81%)	90 (57%)				
Trigone line - n (%)				***	***	0.08	***
No	148 (51%)	24 (13%)	60 (38%)				
Yes, not isolated	40 (14%)	47 (25%)	29 (18%)				
Yes, isolated	104 (36%)	116 (62%)	71 (44%)				
Stress test							
Duration exercise test	8.1 (6.2-10.0)	6.5 (5.0-9.2)	7.0 (5.1-9.0)	***	**	**	2.08
Reason of ending - n (%)				*	**0.02**	**	1.43
None	15 (5%)	4 (2%)	4 (3%)				
Fatigued	231 (80%)	125 (71%)	99 (64%)				
Chest Pain	2 (1%)	2 (1%)	1 (1%)				
Palpitation	-	3 (2%)	2 (1%)				
Leg Pain	17 (6%)	23 (13%)	20 (13%)				
Other	25 (9%)	19 (11%)	29 (19%)				
Ischemia - n (%)	14 (5%)	13 (7%)	8 (5%)	0.49			
Minimal systolic RR interval (mmHg)	124 (111-136)	127 (113-138)	139 (123-152)	***	0.65	***	***

*Numerical variables are reported as median (interquartile range). Categorical variables are reported as number (percentage). Numerical variables were tested using the kruskal-wallis test. If significant, pairwise comparisons were made using the mann-whitney u-test. Categorical variables were tested with the chi-squared test. Both pairwise comparisons were adjusted using the bonferroni adjustment. *:<0.01, **:<0.001, ***:<0.0001. L(I/S)PV: left (inferior/superior) pulmonary vein; R(I/S)PV: right (inferior/superior) pulmonary vein; proBNP: pro-brain natriuretic peptide; ATII: angiotensin II; CRP: C-Reactive protein; TSH: Thyroid-stimulating hormone.*

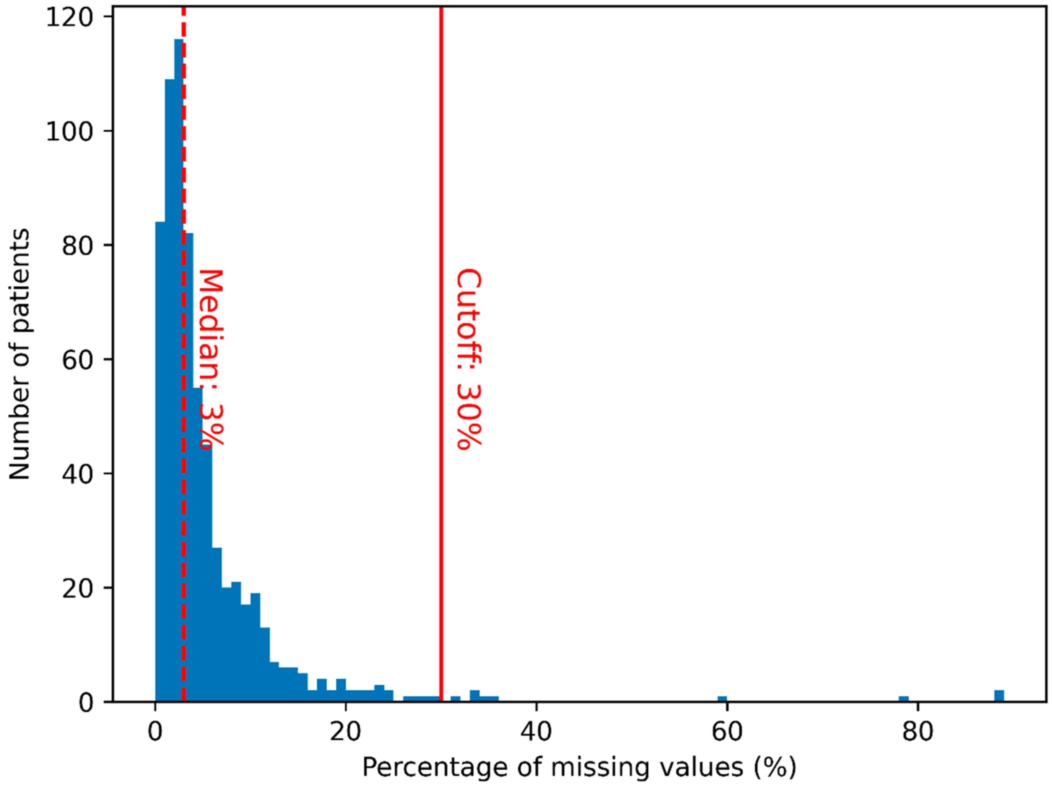

Figure S1: The distribution of the percentage of missing values in each patient after removing the variables that have more than 30% missing values. Patients with more than 30% missing values were excluded. The median of the percentage of missing values was only 3%.

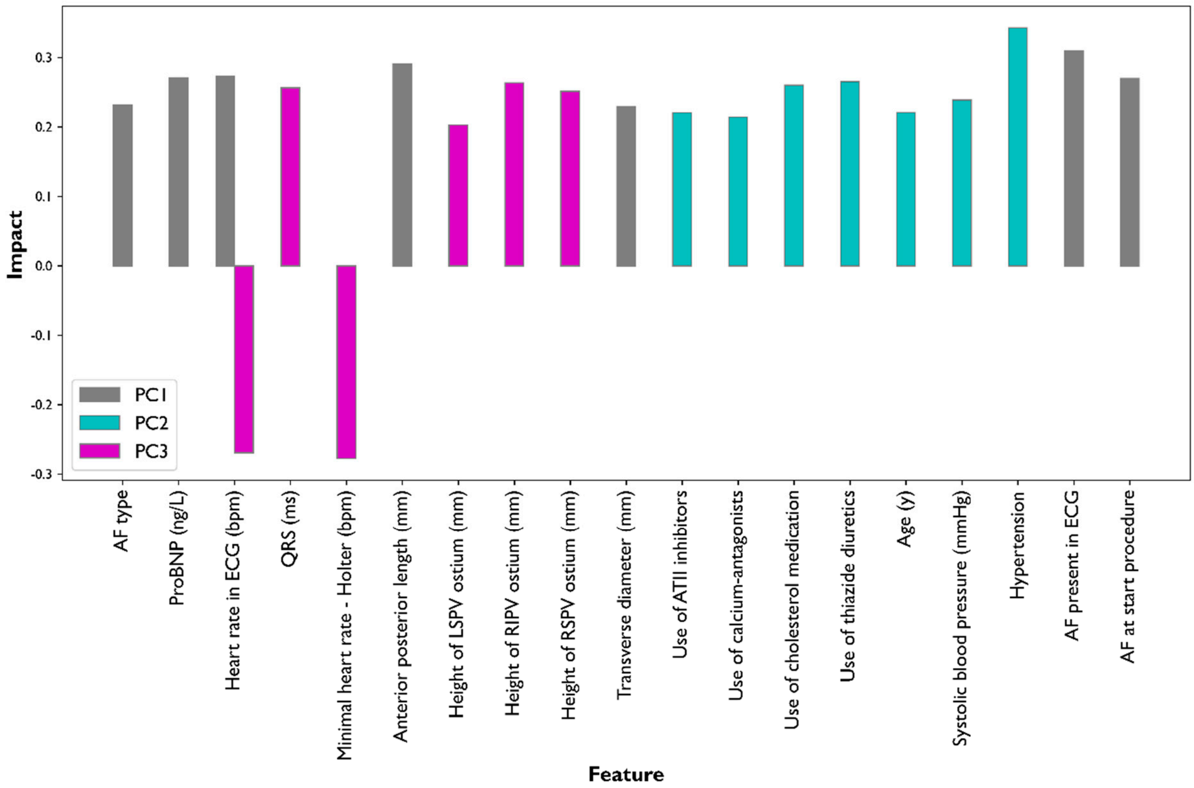

Figure S2: Clinical variables with the highest impact on each principal component. AF: atrial fibrillation; L(I/S)PV: left (inferior/superior) pulmonary vein; R(I/S)PV: right (inferior/superior) pulmonary vein; proBNP: pro-brain natriuretic peptide; ATII: angiotensin II.

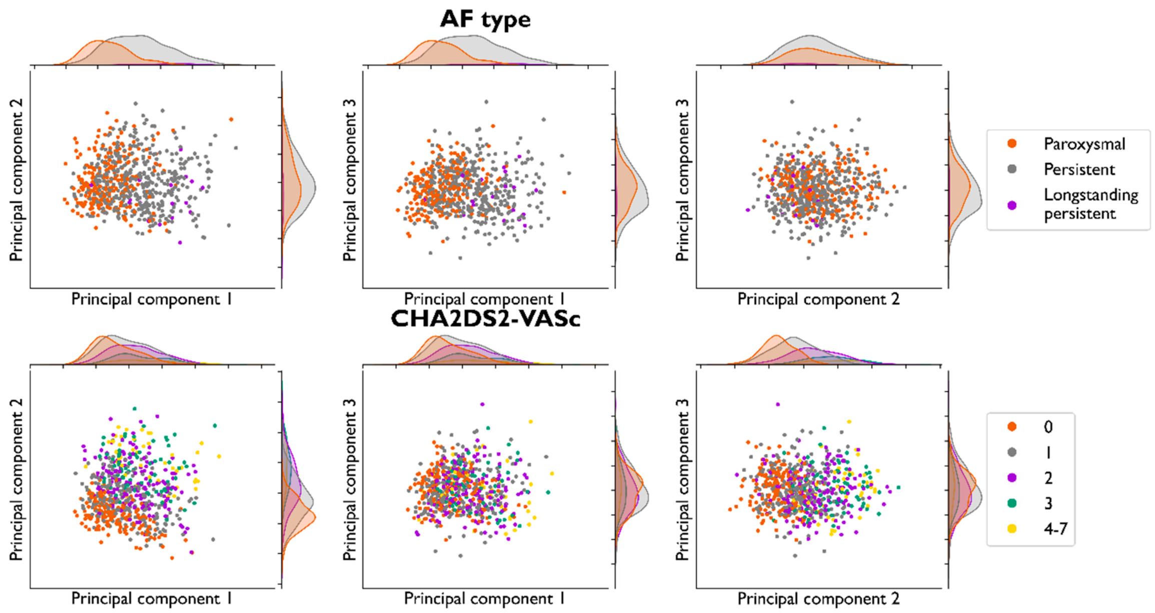

(3%) had longstanding persistent AF, which results in the small distribution curve of longstanding persistent AF patients.

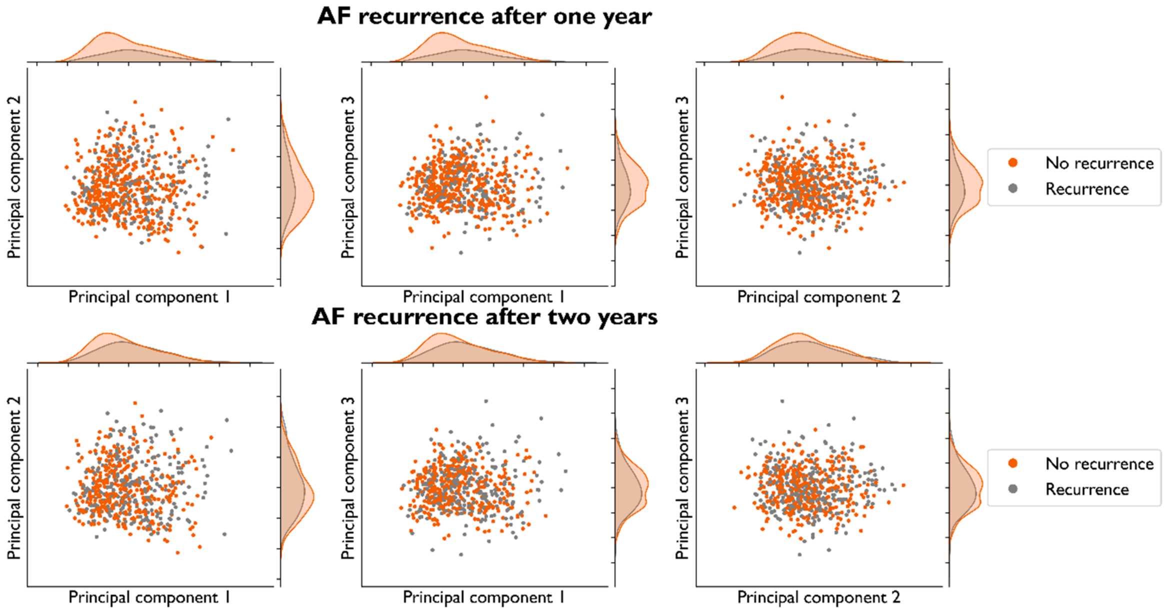

Figure S4: Patients represented as points in each pair of principal components. Each point is colored by the corresponding outcome variable of that patient. The distribution and separation of the outcome variables in each of the principal components can be visually analyzed using the distribution curves on the top and right side of the plot. A-C: 1-year AF recurrence. D-F: 2-year AF recurrence. Outcome variables were not used to build the principal component analysis model.
