# Supplementary figures and images for: Unsupervised Clustering of Patients Undergoing Thoracoscopic Ablation Identifies Relevant Phenotypes for Advanced Atrial Fibrillation
Source: Diagnostics (Basel). 2025 May 16;15(10):1269. doi: 10.3390/diagnostics15101269 (PMC12110638; doi:10.3390/diagnostics15101269)

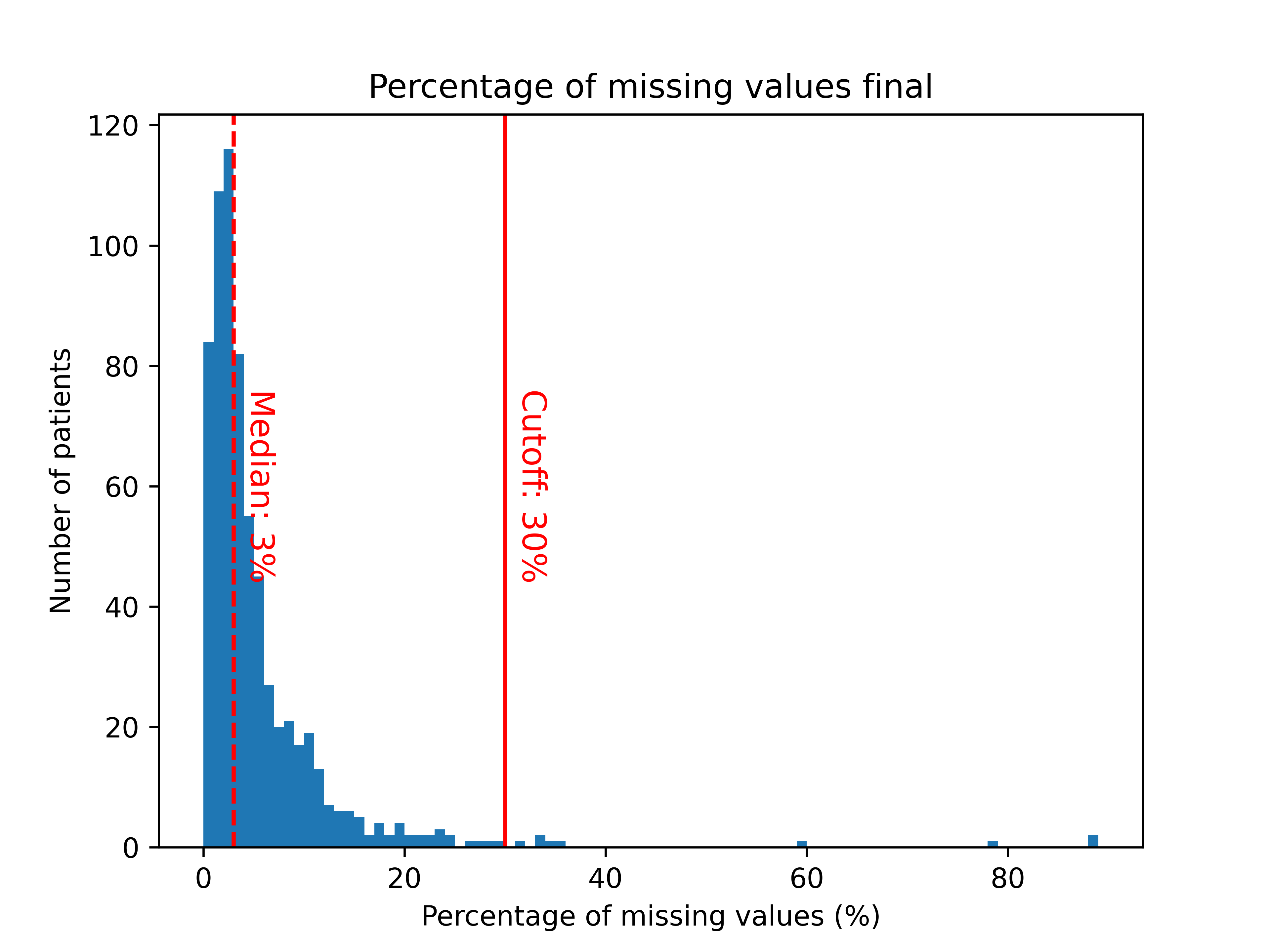

Supplement: Supplementary file 1 [file diagnostics-15-01269-s001.zip › Figure S1.png]

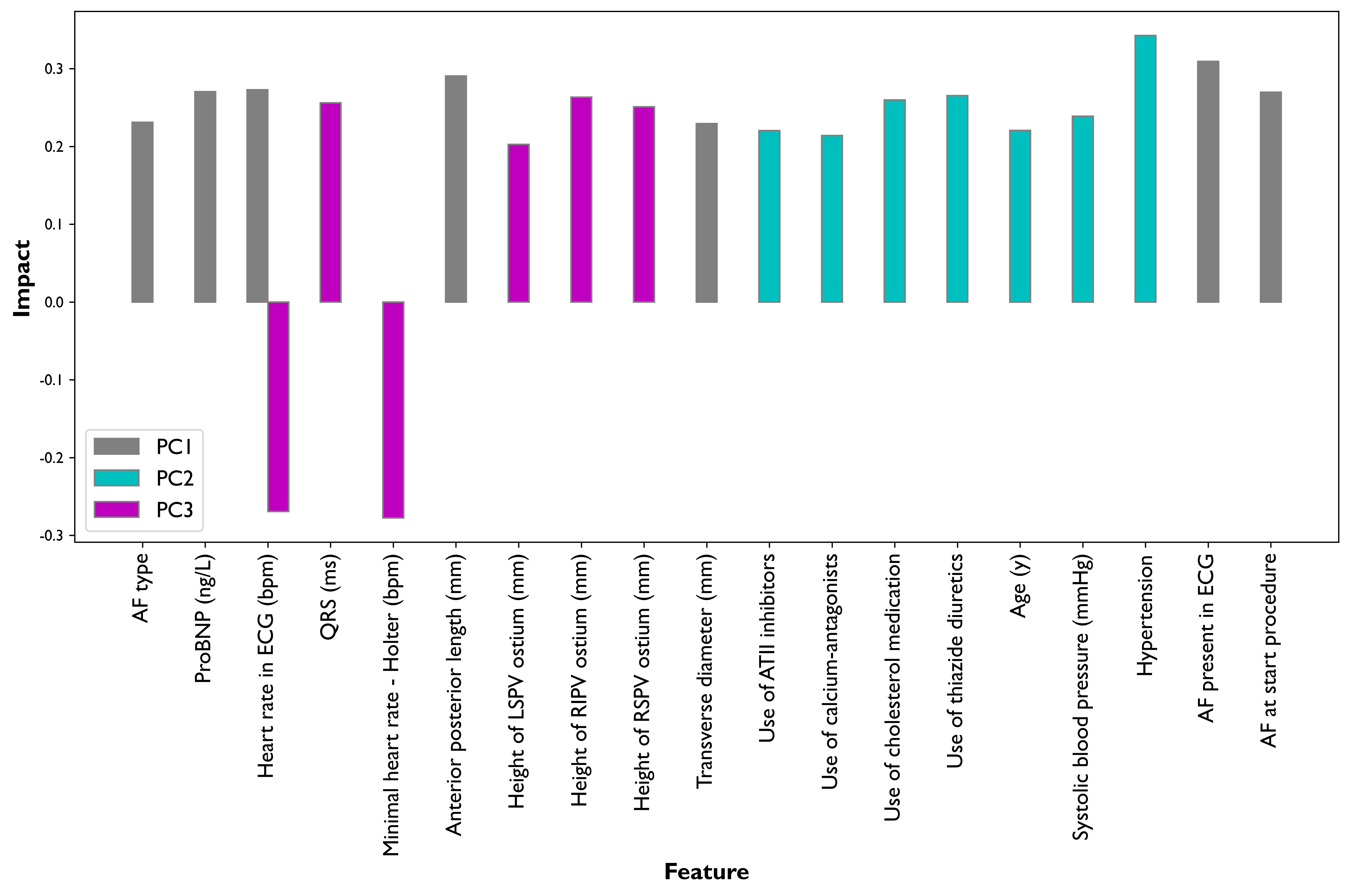

Supplement: Supplementary file 1 [file diagnostics-15-01269-s001.zip › Figure S2.png]

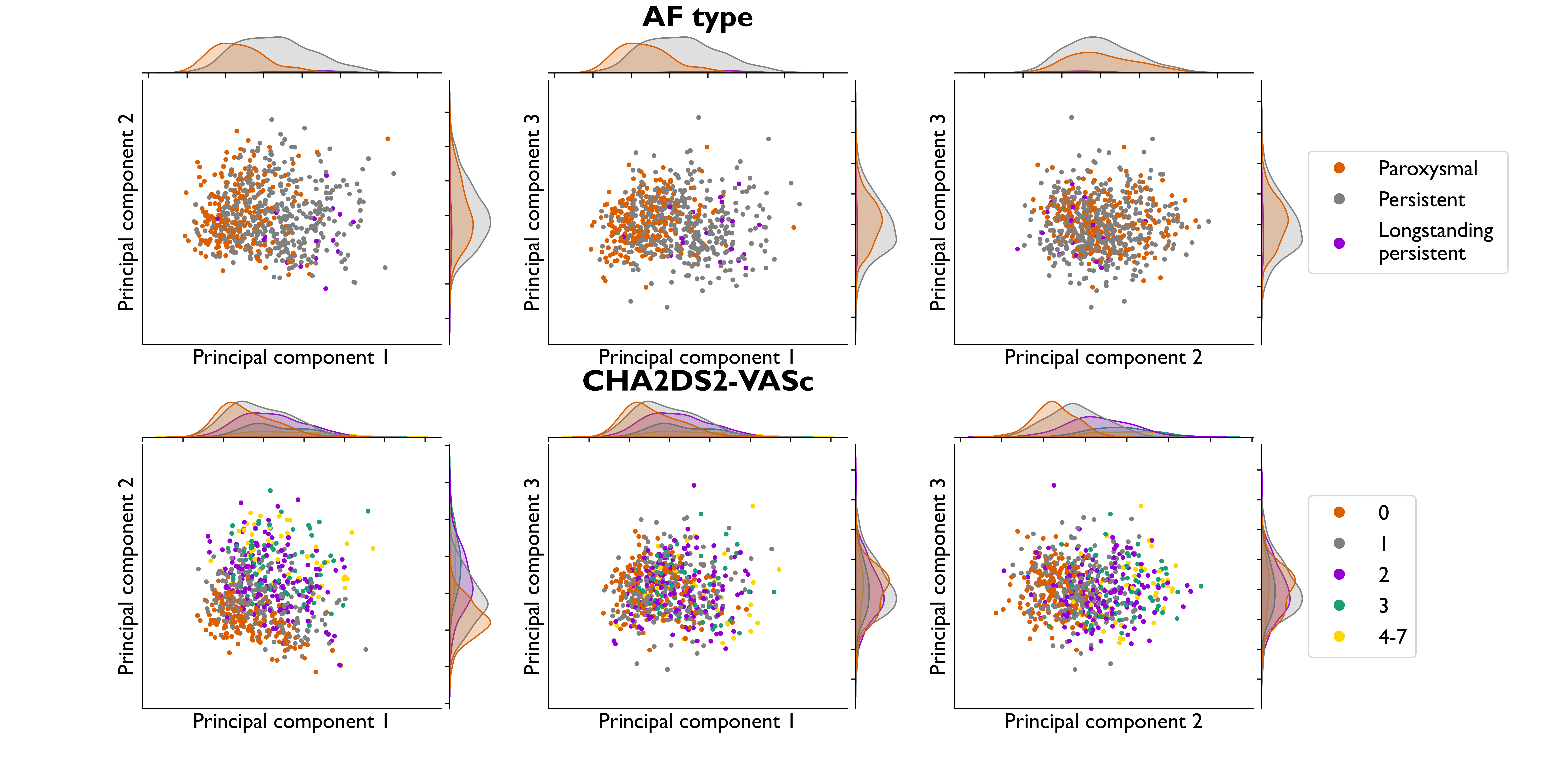

Supplement: Supplementary file 1 [file diagnostics-15-01269-s001.zip › Figure S3.png]

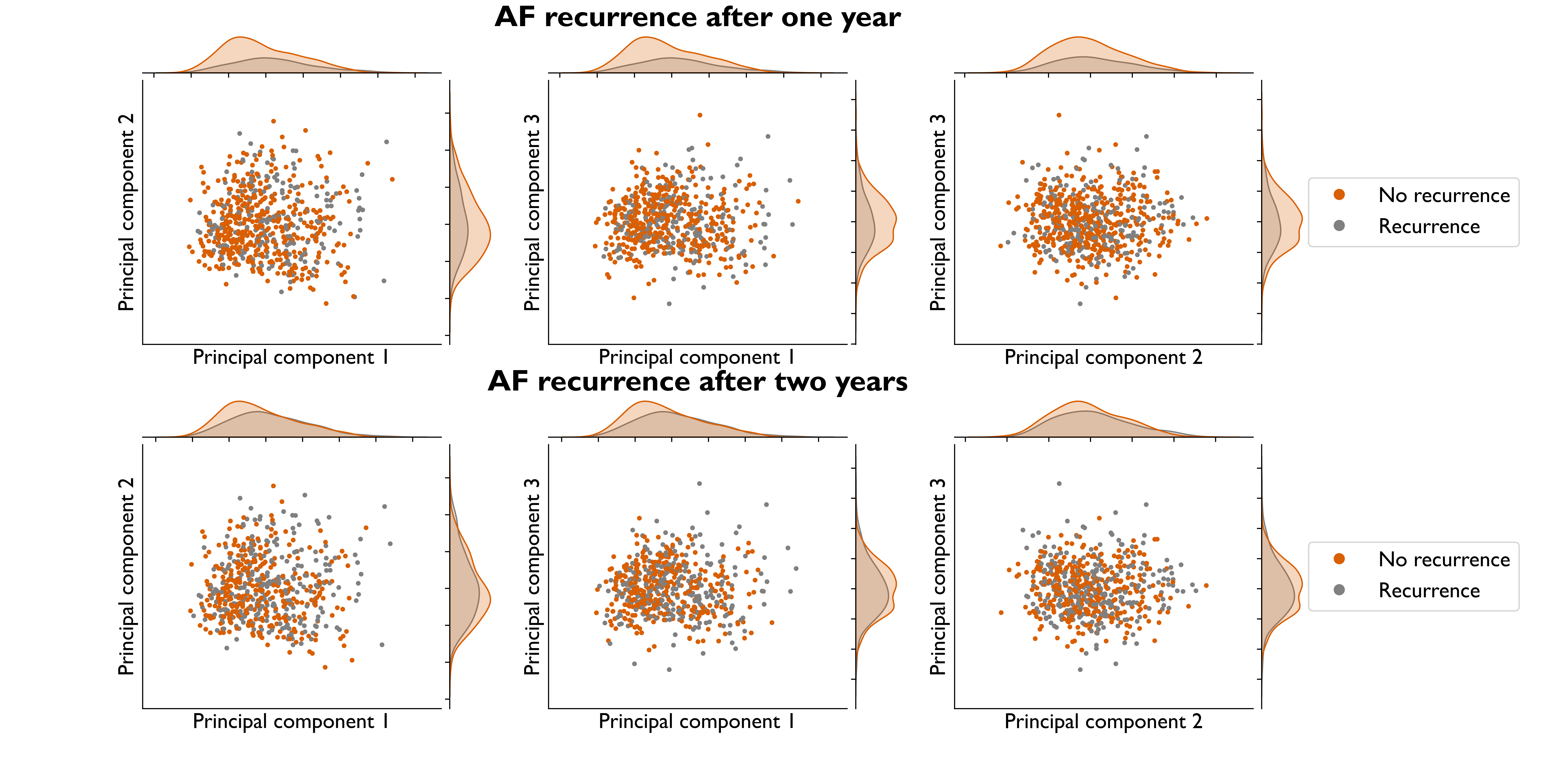

Supplement: Supplementary file 1 [file diagnostics-15-01269-s001.zip › Figure S4.png]
